# Failed Stopping Transiently Suppresses the Electromyogram in Task-Irrelevant Muscles

**DOI:** 10.1523/ENEURO.0166-24.2025

**Published:** 2025-01-28

**Authors:** Isaiah Mills, Mitchell Fisher, Corey George Wadsley, Ian Greenhouse

**Affiliations:** Action Control Lab, Department of Human Physiology, University of Oregon, Eugene, Oregon 97403

**Keywords:** cognitive control, electromyography, motor control, response inhibition, stop signal task

## Abstract

Selectively stopping individual parts of planned or ongoing movements is an everyday motor skill. For example, while walking in public, you may stop yourself from waving at a stranger who you mistook for a friend while continuing to walk. Despite its ubiquity, our ability to selectively stop actions is limited. Canceling one action can delay the execution of other simultaneous actions. This stopping-interference effect on continuing actions during selective stopping may be attributed to a global inhibitory mechanism with widespread effects on the motor system. Previous studies have characterized a transient global reduction in corticomotor excitability by combining brain stimulation with electromyography (EMG). Here, we examined whether global motor inhibition during selective stopping can be measured peripherally and with high temporal resolution using EMG alone. Eighteen participants performed a bimanual anticipatory response inhibition task with their index fingers while maintaining a tonic contraction of the task-irrelevant abductor digiti minimi (ADM) muscles. A time series analysis of the ADM EMG signal revealed transient inhibition during failed stopping compared with go response trials 150 to 203 ms following the stop signal. The pattern was observed in both hands during bimanual stop-all trials as well as selective stop-left and stop-right trials of either hand. These results indicate that tonic muscle activity is sensitive to the effects of global motor suppression even when stopping fails. Therefore, EMG can provide a physiological marker of global motor inhibition to probe the time course and extent of stopping processes.

## Significance Statement

The ability to stop ongoing actions is disrupted in a variety of brain disorders, and failing to stop can have dire consequences for personal safety. Successfully stopping an initiated response has a widespread inhibitory effect on motor system excitability. By continuously measuring activity in task-irrelevant muscles during the performance of a stop task, we unveiled a novel signature of transient motor system inhibition when stopping fails. The pattern was observed during attempts to selectively and nonselectively stop actions. This temporally precise signature of peripheral inhibition may be leveraged to better examine candidate neural mechanisms, and our noninvasive approach is well suited for tracking inhibitory control deficits in clinical populations.

## Introduction

Response inhibition is the process of canceling planned or initiated actions to meet environmental demands. Often, these demands require us to stop only part of our planned actions while continuing the remaining parts. The ability to selectively stop a subset of actions is essential for flexible behavior. In the laboratory, behavioral stopping is often investigated using response inhibition tasks in which participants execute a response associated with a go signal and attempt to cancel this response upon the presentation of a stop signal (Logan et al., 1984). Evidence from many studies using various iterations of stop signal and anticipatory response inhibition (ARI) tasks indicates that stopping globally inhibits the motor system. Specifically, corticomotor excitability (CME) is reduced in both task-relevant and task-irrelevant muscles during stopping, as indexed by decreased motor evoked potential (MEP) amplitudes elicited with transcranial magnetic stimulation (TMS; Badry et al., 2009; Majid et al., 2012). Global inhibition is generalizable, as stopping verbal responses (Cai et al., 2012) and inhibiting eye saccades (Wessel et al., 2013) also reduced CME of task-irrelevant hand muscles.

Global effects of response inhibition on the motor system are also observable during selective stopping, when part of a multieffector response is canceled (Wadsley et al., 2022a). Selective stopping is typically investigated using multicomponent response inhibition paradigms during which a stop-signal is presented for only one effector while the remaining effectors should continue responding as intended. However, the continuing response is frequently delayed during selective stopping, a phenomenon referred to as the stopping-interference effect (Coxon et al., 2007; Aron and Verbruggen, 2008; MacDonald et al., 2012; Wadsley et al., 2019). The stopping-interference effect is attributed to spillover from the global inhibitory stopping mechanism, evident in suppressed CME of the nonstopping effector during the time of response inhibition (MacDonald et al., 2014, Cowie et al., 2016). The magnitude of this effect is sensitive to the degree of functional coupling, as shown by a smaller stopping-interference effect when the prepared effectors are decoupled (Jana and Murthy, 2018; Wadsley et al., 2019, 2022b).

Global inhibitory effects of stopping are typically studied using TMS-derived measures of CME at discrete time points within the estimated time of stopping; however, this methodology has several limitations. First, discrete time points must be determined a priori (Greenhouse et al., 2012), providing poor temporal resolution of response inhibition as it manifests at the effector and requiring repeated sampling over the course of long experiments. Additionally, certain populations of interest are ineligible for TMS (Rossi et al., 2021), complicating the generalizability of effects. In contrast, surface electromyography (EMG) is safe in most populations and offers high temporal resolution. Recent studies highlight the value of EMG for identifying informative physiological markers of stopping including within-trial estimates of response inhibition onset (Jana et al., 2020; Raud et al., 2022). We hypothesized global inhibition during stopping may be detectable in task-irrelevant muscles if they remain tonically active, since patterns in tonic EMG may be sensitive to global task-dependent changes in CME. Therefore, tonic EMG activity of task-irrelevant muscles may offer a more universally applicable approach to measuring the time course, magnitude, and spatial spread of global inhibition during stopping.

In this study, participants completed a bimanual selective stopping ARI task with their index fingers as responding effectors while maintaining tonic contractions with their pinky fingers to test for signatures of inhibition in tonic EMG. The EMG activity in the task-irrelevant pinky muscles was compared across go trials, nonselective stop-all trials, and selective stop trials. We hypothesized tonic EMG amplitude in these task-irrelevant effectors would transiently decrease during successful and failed stopping compared with go trials, owing to the recruitment of a global inhibitory mechanism. Such a pattern of results could provide valuable information about the time course and extent of the global inhibitory process at the level of the muscles with greater sensitivity than commonly derived behavioral stopping and TMS measurements.

## Materials and Methods

### Participants

Twenty participants provided informed consent in accordance with the University of Oregon institutional review board. Potential participants with a history of specific movement disorders were excluded from the study. Out of the 20 initial participants, one participant was excluded for poor task performance (<25% successful stopping accuracy), and another was excluded for the inability to maintain tonic contractions, which left 18 datasets (nine female, age = 21.6 ± 1.1, all self-reported right handed) for analysis.

### ARI task

**Figure 1.**
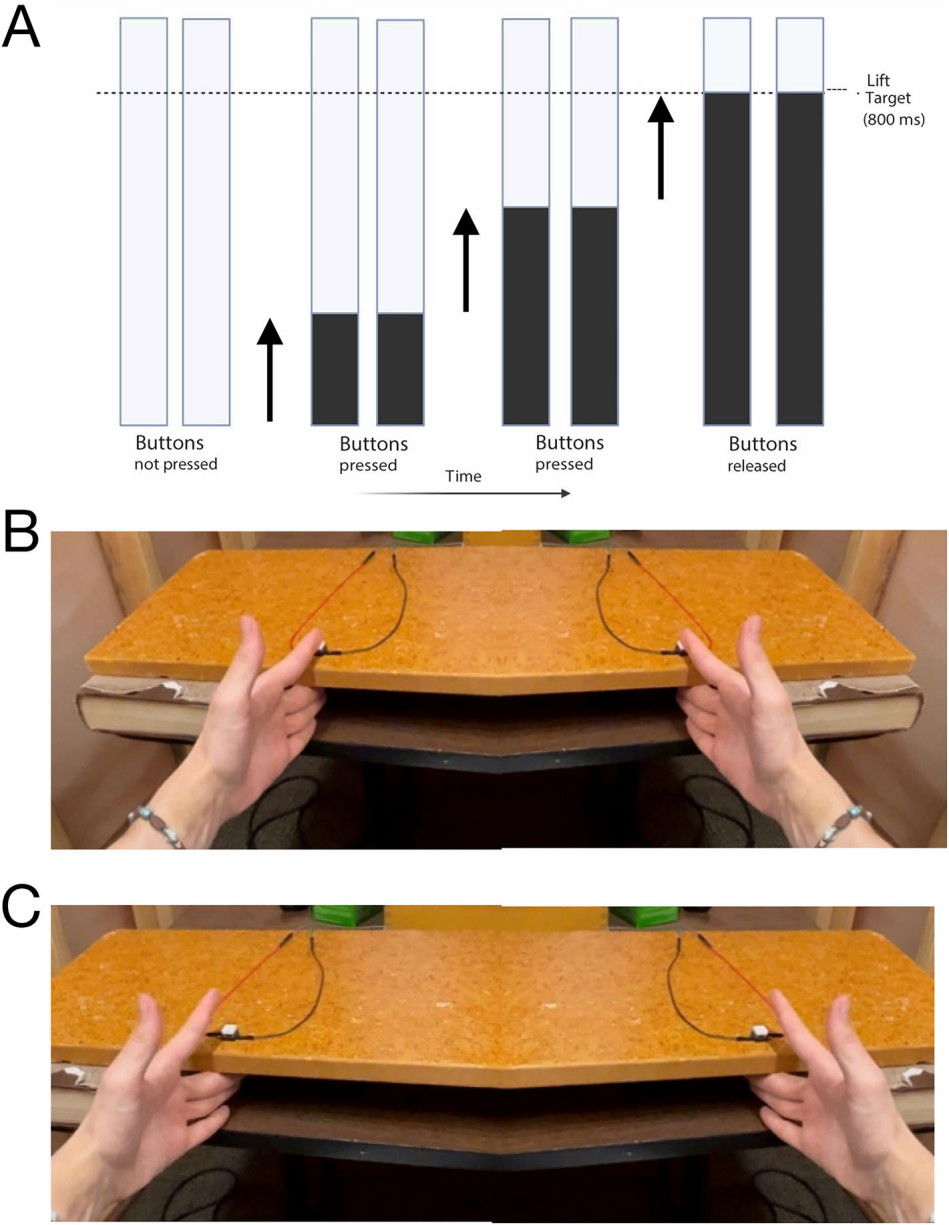
***A***, Stimuli displayed on a computer monitor consisted of two vertical bars that filled from the bottom to the top over the course of a 1 s interval. Participants were instructed to time their responses with when the filling bars intersected a response target line. ***B***, Participants tonically contracted the ADM by pressing their pinky fingers downward onto the table surface while pressing both response buttons with their index fingers. ***C***, Participants made responses by lifting their index fingers off of the response buttons while maintaining the tonic contraction in the pinky fingers.

We investigated the effects of the stopping process using a bimanual ARI task, coded in MATLAB 2019a (Fig. 1). Participants sat ∼1 m from a computer screen (refresh rate, 60 Hz) with their arms supported on armrests, hands extended, and palms facing toward the sagittal midline. The third, fourth, and fifth digits of each hand rested on the surface of the table in front of them in the space between the table and a raised wooden platform of adjustable height. Response buttons were positioned on the elevated platform beneath the medial surface of the left and right index fingers. The response buttons were button switches wired to a MakeyMakey® (Joylabs) connected to the task computer. Participants pressed their pinky fingers downward against the table through abduction, away from the palm, for the complete duration of all task trials.

The default task display began with two white parallel vertical bars on a gray background. Each task trial started when both buttons on the elevated platform were pressed simultaneously with the index fingers which triggered the bars to begin filling from bottom to top. The bars took 1 s to fill completely. Participants were instructed to time the lifting of their fingers off of the buttons to the intersection of the filling bars with the target line (800 ms from trial onset, Fig. 1). The subsequent trial started 1 s after the filling bar reached the target line. Two-thirds of trials were go trials, during which the left and right bars filled for as long as their corresponding button was pressed, and stopped filling only when the corresponding button was released, i.e., the index fingers were lifted off the buttons.

The remaining one-third of trials were an equal mixture of stop-both, stop-left, and stop-right trials. During stop-both trials, both bars stopped filling automatically after a predetermined stop signal delay (SSD). Participants were instructed to cancel both of their planned index finger lifts while maintaining the contraction with their pinky fingers when the bars stopped. The SSD in this experiment refers to the difference between the target response time and the presentation of the stop signal, and as such is reported as a negative value, where more negative values indicate more time available for stopping. During selective stop-left and stop-right trials, one bar stopped filling at a separate predetermined SSD, while the other bar continued filling to the target. On selective stop trials, participants were instructed to cancel the lift of the finger corresponding to the stopped bar while attempting to lift the remaining finger when its respective bar reached the target line.

**Figure 2.**
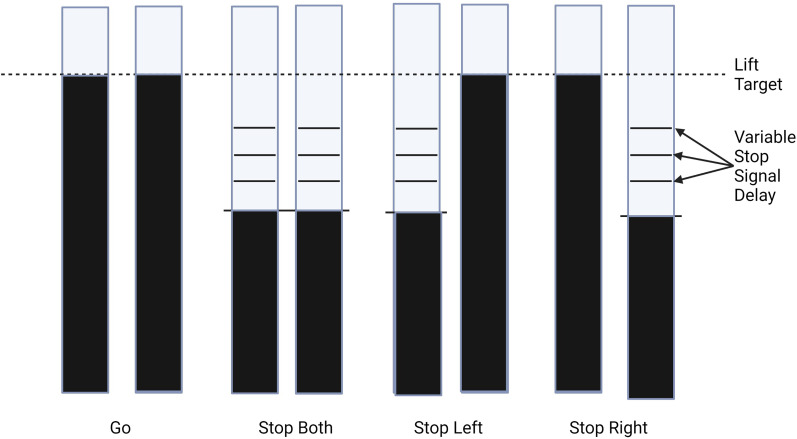
Example task stimuli are shown with each pair of bars corresponding to one of the four types of trials: go, stop-both, selective stop-left, and selective stop-right, respectively. Participants were instructed to lift both index fingers when the bars crossed the target line. If a bar stopped before the response target, participants attempted to cancel the finger lift(s) on the side corresponding to the stopped bar(s).

SSDs started at −200 ms for stop-both trials and −250 ms for selective stop-left and stop-right trials. Initial SSD values were based on the expected time required for nonselective and selective stopping in healthy young adults (MacDonald et al., 2012). SSDs were adjusted individually for each stop trial type using a staircase procedure (Fig. 2). The use of independent SSD staircases was chosen to attain a 50% probability of successful stopping for each stop trial type. The SSD of a particular stop trial type decreased by 50 ms after a failed stop trial (when participants lifted their fingers despite the bar stopping) and increased by 50 ms after a successful stop trial, corresponding to more and less time to stop following failed and successful stop trials, respectively. When participants correctly inhibited a response during a stop trial or lifted the correct buttons within 50 ms of the target line during a go or selective stop trial, feedback was given in the form of the response target line turning green. If the participant responded between 50 and 100 ms of the target line, the response target line turned yellow. Finally, if a response was recorded during a stop trial or outside the response window (< or >100 ms of the response target), the target line turned red.

Participants completed 9 blocks of 32 trials (192 go, 32 stop-both, 32 stop-left, 32 stop-right; 288 trials total), and the order of trial types was randomized for each participant. After each block, participants received feedback about their performance in that block, including their average response time relative to the target line. Participants were encouraged to take breaks between task blocks and instructed to press both buttons when ready to continue.

### Behavioral metrics

Response time was calculated for all trials by determining the difference between the index finger response time and the target response time (0.8 s). Negative values reflect responses prior to the target and positive values reflect responses after the target. Lift accuracy was calculated for go and selective stopping trials as the proportion of trials in which the participants responded within 100 ms of the target response time. Stop trial accuracy was calculated as the proportion of trials where the response was successfully withheld. Stop signal reaction time (SSRT), an estimate of the latency of the stopping process, was calculated using the integration method with the replacement of go omissions (Verbruggen et al., 2019) separately for the three different types of stop trials. The stopping-interference effect was calculated for the left hand by subtracting the mean response time of the left hand during go trials from the mean response time of the left hand during stop-right trials. The same procedure was employed to calculate the interference effect in the right hand during stop-left trials.

### Electromyography

We recorded surface EMG using bipolar electrodes adhered to the skin above the first dorsal interosseous (FDI) muscle and the abductor digiti minimi (ADM) of both hands. A ground electrode was attached above the styloid process of the left ulna. EMG was sampled at 5,000 Hz, amplified by a factor of 1,000, and bandpass filtered (50–450 Hz; Delsys). Visualization and analysis of EMG data were performed using the VETA toolbox for MATLAB (Jackson and Greenhouse, 2019). Prior to beginning data collection, participants performed a maximum voluntary contraction (MVC) of each ADM to assess their maximum contractile output. This was done by computing the average maximum EMG amplitude measured during four consecutive 1 s contractions with both pinky fingers. Each participant was instructed to maintain a tonic contraction at 10% of their MVC during the task. Experimenters observed online EMG data traces on a separate monitor positioned adjacent to the stimulus display in order to monitor participants’ contraction and data quality. EMG data was recorded for the duration of each individual trial as well as for 1 s after each trial concluded. Participants completed a set of practice trials (40 trials, 10 of each type) with EMG recording prior to starting the experiment to gain familiarity with the task and to assess EMG data quality and were allowed to repeat the practice block until they felt confident in completing the task correctly.

For each participant, ADM EMG data were rectified and *z*-scored on a trial-by-trial basis and then averaged across trials for each trial type generating 1.8 s duration mean EMG traces for go, stop-both, stop-left, and stop-right trials. Baseline EMG amplitude was calculated as the average tonic EMG amplitude of the 500 ms preceding the stop signal. Past studies using TMS in the ARI paradigm have compared relevant MEPs to a baseline MEP at −600 ms (MacDonald et al., 2014) up to −175 ms relative to target response time (Cowie et al., 2016). In both studies, the anticipatory nature of the task was not assumed to affect the integrity of the chosen baseline. The *z*-scored data of each participant was then rectified across a moving window of 12 sample points, equivalent to a 2.4 ms window (Fisher et al., 2023). Comparisons of EMG across go trials and successful and failed stop trials of all types were performed separately for the left and right ADM. This was done by locking individual trial EMG to stop-signal onset during stop trials and the average SSD on go trials (Jana et al., 2020). Specifically, a 600 ms epoch of interest was defined that started 100 ms before each individual trial’s stop signal for stop trials, and 100 ms before the average SSD for go trials. The duration of this epoch ensured the entire period of the estimated SSRTs was included.

Mean FDI EMG onsets and peak amplitudes were calculated relative to the stop signal for each hand during go trials, failed stop, and successful stop trials of all types and are reported in Table 2. Onset times were determined using the VETA toolbox “findEMG.m” function (Jackson and Greenhouse, 2019). In brief, the FDI EMG onset times were determined to be the first time points at which the rectified EMG signal exceeded two standard deviations of the mean within a trial.

To check for possible effects of fatigue, we calculated the mean root mean square (RMS) of the ADM EMG signal during the baseline epoch averaged across each block.

### Statistical analyses

We tested for differences in SSRT and stop accuracy across stop trial types using a 1 × 3 (stop-both, stop-left, stop-right) repeated measures (RM) ANOVA with Bonferroni’s corrected post hoc paired *t* tests. We tested for differences in response times and response accuracies using a 2 × 3 RM ANOVA with the factors hand (left, right) and trial type (go, failed stop-both, selective stop response). The magnitude of the stopping-interference effects during selective stop trials was compared between hands using a paired sample *t* test.

To test for suppression of the tonic EMG signal during the stopping epoch, time series analyses were conducted using pairwise *t* tests at each of 3,000 time points of interest across trial types. These time points correspond to the previously mentioned 600 ms epoch. A false discovery rate (FDR) correction with an adjusted alpha level of 5% was used to account for multiple comparisons in each case (Benjamini and Hochberg, 1995). Specific comparisons of interest included go versus successful stop-both, go versus successful stop-left, and go versus successful stop-right trials. Additional analyses compared failed stopping between failed stop-both, failed stop-left, and failed stop-right trials using the same approach. Data from the left and right ADM were analyzed separately in all cases.

To test for differences in background ADM EMG across blocks, we conducted a one-way RM ANOVA of the mean RMS ADM EMG. Additionally, post hoc, we calculated the statistical power for the comparison of bimanual go versus failed stop-both using the mean ADM EMG activity within the time range of the significant group-level difference to serve as a benchmark for future studies.

### Data and code accessibility

All data and analysis code are available for download via Open Science Framework (osf.io/gkeb3).

## Results

### Behavioral data

Behavioral metrics are presented in Table 1 as mean ± standard deviation. High go accuracy in conjunction with stopping accuracies close to 50% across all stop trial types indicated that participants performed the task correctly and did not adjust their strategies to anticipate stop signals. A 1 × 3 RM ANOVA showed a significant main effect of trial type for SSRT [*F*_(2,17)_ = 3.39; *p* = 0.03; *η*^2^ = 0.223]. Mauchly’s test indicated that the assumption of sphericity had been met [*χ*^2^_(2,17)_ = 2.55; *p* = 0.28]. Post hoc *t* tests showed stop-right SSRT values were significantly longer than stop-both SSRTs (*t* = 2.62; *p* < 0.01), and stop-left SSRTs were longer than stop-both SSRTs (*t* = 2.43; *p* = 0.01). The difference between stop-left and stop-right SSRTs did not reach significance (*t* = 0.34; *p* = 0.37). A 1 × 3 RM ANOVA showed no effect of trial type on stopping accuracy for the stop-both, stop-right, and stop-left conditions [*F*_(2,17)_ = 0.52; *p* = 0.60]. Mauchly’s test indicated that the assumption of sphericity had been met [*χ*^2^_(2,17)_ = 0.51; *p* = 0.775].

**Table 1.** Behavioral metrics of interest mean ± std

Trial type	Go left	Go right	Stop-both	Stop-left	Stop-right
Response time (ms)	16 ± 5	12 ± 5		111 ± 29	106 ± 32
Interference effect (ms)				95 ± 27	94 ± 33
Failed stop RT (ms)			−11 ± 8	−15 ± 26	−3 ± 28
SSRTs (ms)			287 ± 52	310 ± 46	314 ± 47
SSD (ms)			−237 ± 45	−258 ± 38	−262 ± 55
Stop accuracy (%)			48 ± 2	50 ± 2	49 ± 3
Lift accuracy (%)*	88 ± 6	91 ± 5		49 ± 16	51 ± 21
Failed stop trial lift accuracy (%)*			98 ± 2	86 ± 11	84 ± 17

Abbreviations: RT, response time; SSRT, stop signal reaction time; SSD, stop signal delay. * indicates proportion of responses ±100 ms of lift target.

A 2 × 3 RM ANOVA with the factors hand (left, right) and trial type (go, failed stop-both, selective stop response) showed no difference in response time for the main effect of hand [*F*_(1,17)_ = 0.13; *p* = 0.72], and a main effect of trial type [*F*_(2,17)_ = 164.7; *p* < 0.001; *η*^2^ = 0.83], with no interaction between factors [*F*_(2,17)_ = 0.3; *p* = 0.74]. Mauchly’s test indicated that the assumption of sphericity had been met [*χ*^2^_(2,17)_ = 5.44; *p* = 0.066]. Post hoc analyses revealed that stop-right response times (left index responses) were significantly longer than go trial left-hand response times (*t* = 14.3; *p* < 0.001) and failed stop-both left-hand response times (*t* = 12.7; *p* < 0.001). Stop-left response times (right index responses) were significantly longer than go trial right-hand response times (*t* = 11.4; *p* < 0.001), and failed stop-both right-hand response times (*t* = 8.8; *p* < 0.001). Right-hand failed stop-both responses were shorter than go responses (*t* = 4.6; *p* < 0.001), but the difference between left-hand failed stop-both responses and go responses did not reach significance after correcting for multiple comparisons (*t* = 2.1; *p* < 0.05; uncorrected). There was no difference in the magnitude of the stopping-interference effect between hands (*t* = 1.96; *p* = 0.07).

The majority of lifts occurred within ±100 ms of the target, corresponding to the majority of responses falling within this window for go trials (≥88%) and failed stop trials (≥84%). Lift accuracies were lower among responding hands during successful selective stop trials (≥49%), but this was an expected manifestation of the interference effect. A 2 × 3 RM ANOVA was conducted to determine the effects of hand (left, right) and trial type (go, failed stop-both, selective stop response) on response accuracy. Mauchly’s test indicated that the assumption of sphericity had been violated [*χ*^2^_(2,17)_ = 26.12; *p* < 0.01], so degrees of freedom were adjusted using the Greenhouse–Geisser method (ε = 0.55). The ANOVA revealed a significant effect of trial type on lift accuracies [*F*_(1.11,17)_ = 167.9; *p* < 0.001; *η*^2^ = 0.81], with no difference between hands [*F*_(1,17)_ = 0.13; *p* = 0.72], and no interaction between factors [*F*_(1.03,17)_ = 0.09; *p* = 0.79]. Post hoc *t* tests showed that right-hand selective stop response lift accuracy was lower than go (*t* = 9.1; *p* < 0.001) and failed stop-both lift accuracy (*t* = 9.7; *p* < 0.001). Left-hand selective stop response lift accuracy was lower than go (*t* = 8.4; *p* < 0.001) and failed stop-both lift accuracy *(t* = 9.1; *p* < 0.001). There was no difference in lift accuracy between failed stop-both and go trials in the left (*t* = 0.1; *p* = 0.47) or the right hand (*t* = 0.2; *p* = 0.41).

### EMG data

FDI EMG onsets and peak amplitudes were consistent with the behavioral measures and are presented in Table 2. Of note, FDI EMG onset and peak times were similar to those found in the ADM EMG data when comparing go and failed stop trials, although the nature of the task design led to relatively few partial responses (stop-both, 4.78 ± 4.12; stop-left, 4.06 ± 3.61; stop-right, 4.44 ± 4.26) being recorded for each stop trial type. There was a significant difference in baseline mean RMS ADM EMG across blocks [*F*_(8,17)_ = 24.4; *p* < 0.01; *η*^2^ = 0.59]. Post hoc *t* tests showed that baseline mean RMS ADM EMG was greater during block 1 than during block 2 (*t* = 4.99; *p* < 0.001), block 3 (*t* = 8.18; *p* < 0.001), block 4 (*t* = 9.05; *p* < 0.001), block 5 (*t* = 9.49; *p* < 0.001), block 6 (*t* = 9.91; *p* < 0.001), block 7 (*t* = 10.27; *p* < 0.001), block 8 (*t* = 10.45; *p* < 0.001), and block 9 (*t* = 10.52; *p* < 0.001). Baseline mean RMS ADM EMG was greater during block 2 than during block 4 (*t* = 4.08; *p* < 0.01), block 5 (*t* = 4.52; *p* < 0.001), block 6 (*t* = 4.94; *p* < 0.001), block 7 (*t* = 5.30; *p* < 0.001), block 8 (*t* = 5.49; *p* < 0.001), and block 9 (*t* = 5.55; *p* < 0.001). There was no difference in baseline mean RMS ADM EMG between any other blocks. This pattern is consistent with a general decrease in ADM EMG activity over the course of the experiment that leveled off around block 3.

**Table 2.** FDI EMG event times relative to the stop signal (mean SSD for go trials) and number of partial responses (mean ± std)

Trial type	Go	FSB	FSL	FSR	SSB	SSL	SSR
Left FDI EMG onset (ms)	114 ± 14	44 ± 26	108 ± 33	148 ± 32	110 ± 31*	166 ± 34*	263 ± 36
Left FDI EMG peak (ms)	188 ± 15	142 ± 24	213 ± 55	253 ± 47	142 ± 34*^c^	205 ± 73*^c^	352 ± 56
Left FDI partial responses (#)					4.78 ± 4.12	4.06 ± 3.61	
Right FDI EMG onset (ms)	111 ± 17	49 ± 25	103 ± 21	143 ± 25	107 ± 37*	224 ± 41	203 ± 55*
Right FDI EMG peak (ms)	178 ± 20	129 ± 32	174 ± 42	252 ± 53	138 ± 35*^c^	300 ± 46	380 ± 87*^c^
Right FDI partial responses					4.78 ± 4.12		4.44 ± 4.26

Abbreviations: FSB, failed stop-both; FSL, failed stop-left; FSR, failed stop-right; SSB, successful stop-both; SSL, successful stop-left; SSR, successful stop-right. * indicates partial response trials and ^c^ cancel time.

**Figure 3.**
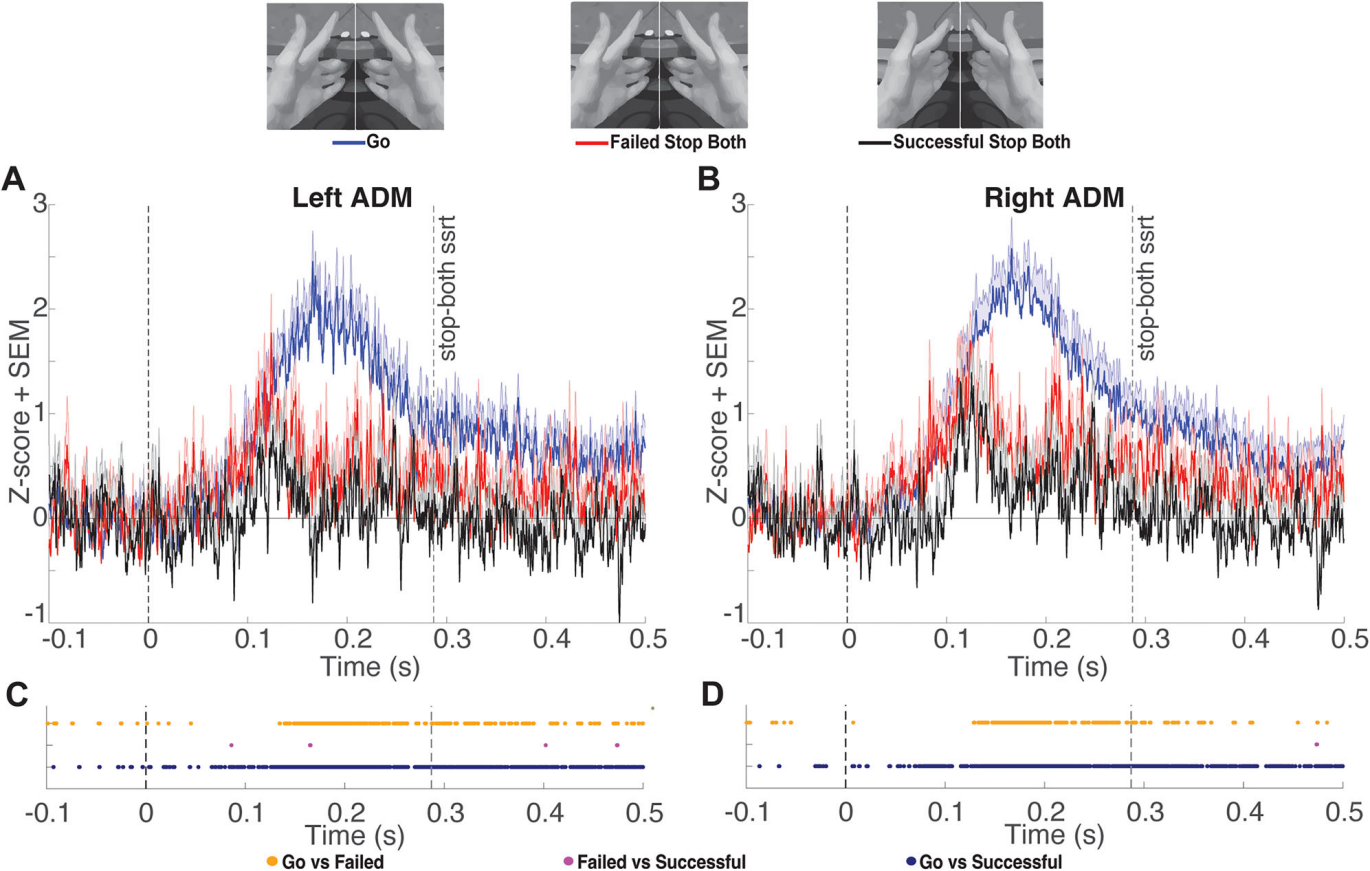
***A***, *Z*-scored tonic left ADM EMG amplitude [mean + standard error of the mean (SEM)] comparing go trials locked to the mean SSD and stop-both trials locked to the stop signal at time point zero. Group mean SSRT is denoted by the rightmost vertical dashed line. ***B***, Same as ***A*** for the right ADM. ***C***, Significant FDR-corrected comparisons (*p* *<* 0.05) for all time points of interest for the left ADM. ***D***, Same as ***C*** for the right ADM.

**Figure 4.**
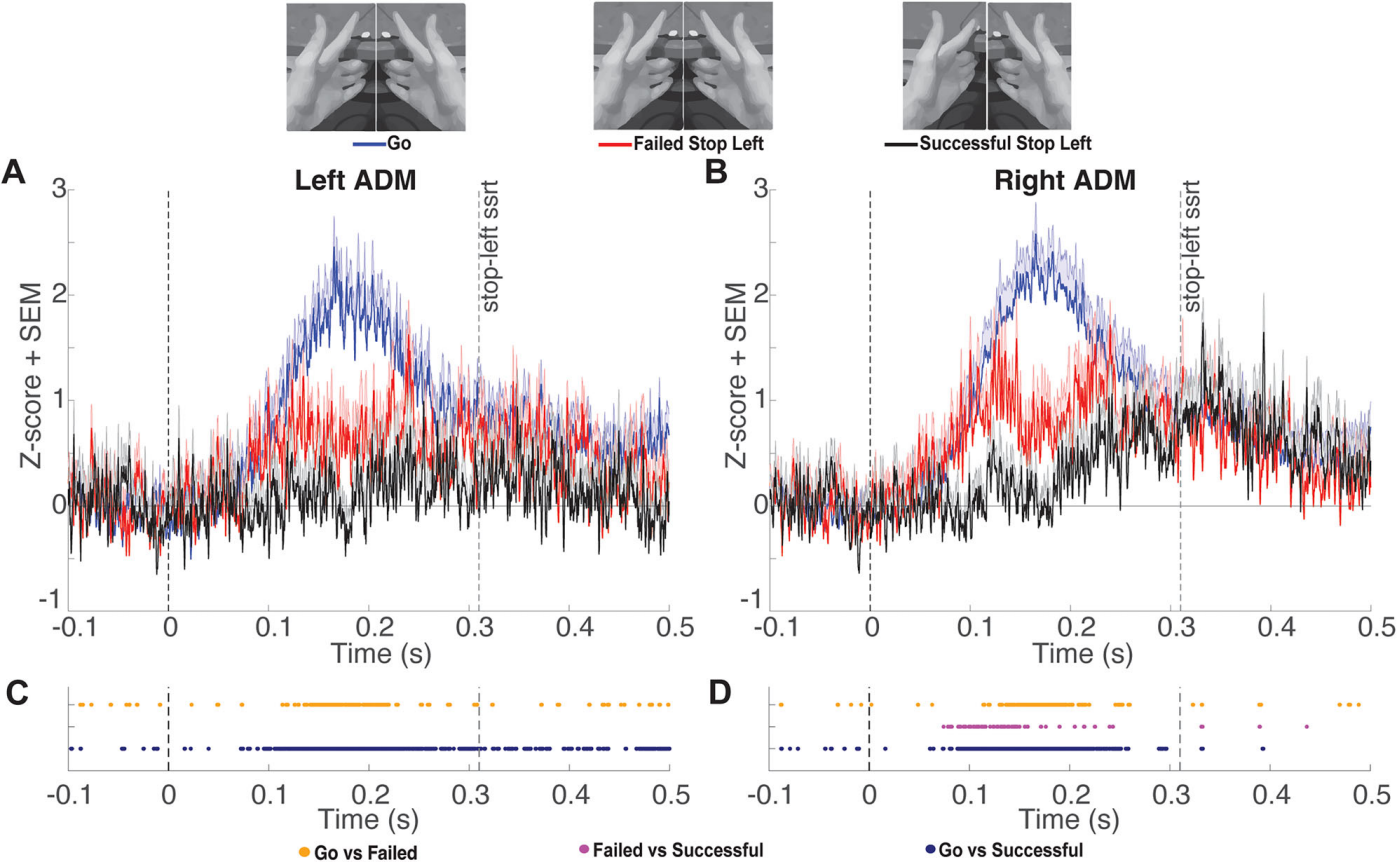
***A***, *Z*-scored tonic left ADM EMG amplitude (mean + SEM) comparing go trials locked to the mean SSD and stop-right trials locked to the stop signal at time point zero. Group mean SSRT is denoted by the rightmost vertical dashed line. ***B***, Same as ***A*** for the right ADM. ***C***, Significant FDR-corrected comparisons (*p* *<* 0.05) for all time points of interest for the left ADM. ***D***. Same as ***C*** for the right ADM.

**Figure 5.**
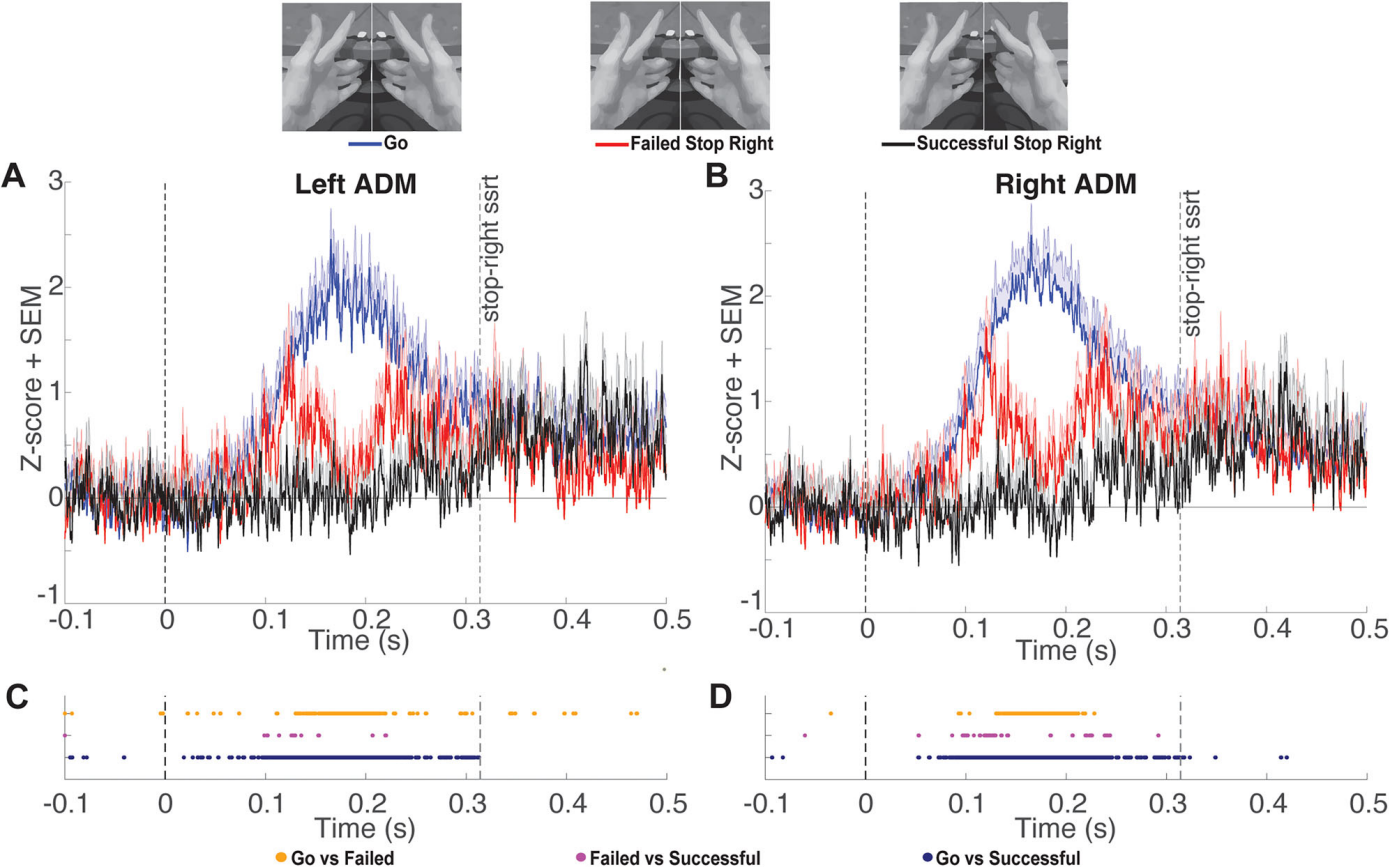
***A***, *Z*-scored tonic left ADM EMG amplitude (mean + SEM) comparing go trials locked to the mean SSD and stop-left trials locked to the stop signal at time point zero. Group mean SSRT is denoted by the rightmost vertical dashed line. ***B***, Same as ***A*** for the right ADM. ***C***, Significant FDR-corrected comparisons (*p* *<* 0.05) for all time points of interest for the left ADM. ***D***, Same as ***C*** for the right ADM.

**Figure 6.**
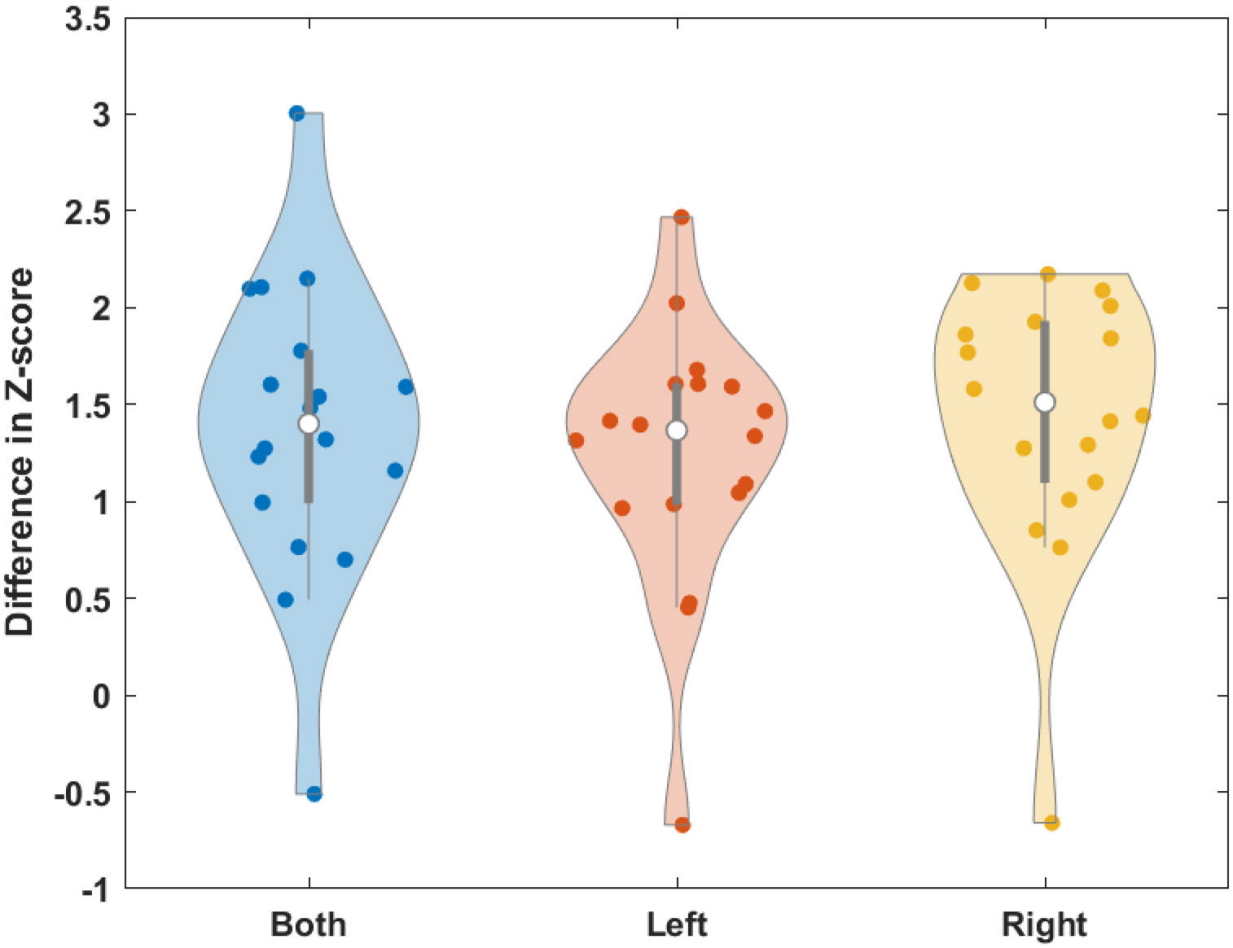
Mean go versus. failed stop *z*-score differences in ADM EMG activity averaged across the two hands during significant epochs identified in Figures 3–5 for stop-both, stop-left, and stop-right, respectively.

Tonic ADM EMG amplitudes during failed stop-both, failed stop-left, and failed stop-right trials exhibited an interval of divergence from go trials characterized by a clear transient dip. To determine the onset and offset of this interval for each type of failed stop trial in each hand, we conducted a post hoc analysis. We identified the longest interval in which >90% of time points showed a significant (FDR corrected) difference between failed stop and go trials. During stop-both trials, the left ADM exhibited significant decreases in tonic EMG output as compared with go trials from 137 to 207 ms following the stop signal, while the right ADM exhibited significant decreases in tonic EMG output from 138 to 205 ms (Fig. 3). During stop-left trials, the left ADM exhibited significant decreases in tonic EMG output from 137 to 231 ms, while the right ADM exhibited significant decreases in tonic EMG output from 137 to 204 ms (Fig. 4). During stop-right trials, the left ADM exhibited significant decreases in tonic EMG output from 140 to 210 ms, while the right ADM exhibited significant decreases in tonic EMG output from 139 to 216 ms (Fig. 5). Overall, these results describe an average period of suppression during failed stopping as compared with go trials from 138 to 212 ms. The difference in ADM EMG activity between go and failed stop trials was present in the majority of participants (Fig. 6). The calculated power for this comparison averaged across hands was 0.95, indicating that our sample was sufficient for detecting a Cohen’s *d* effect size of 0.93.

**Figure 7.**
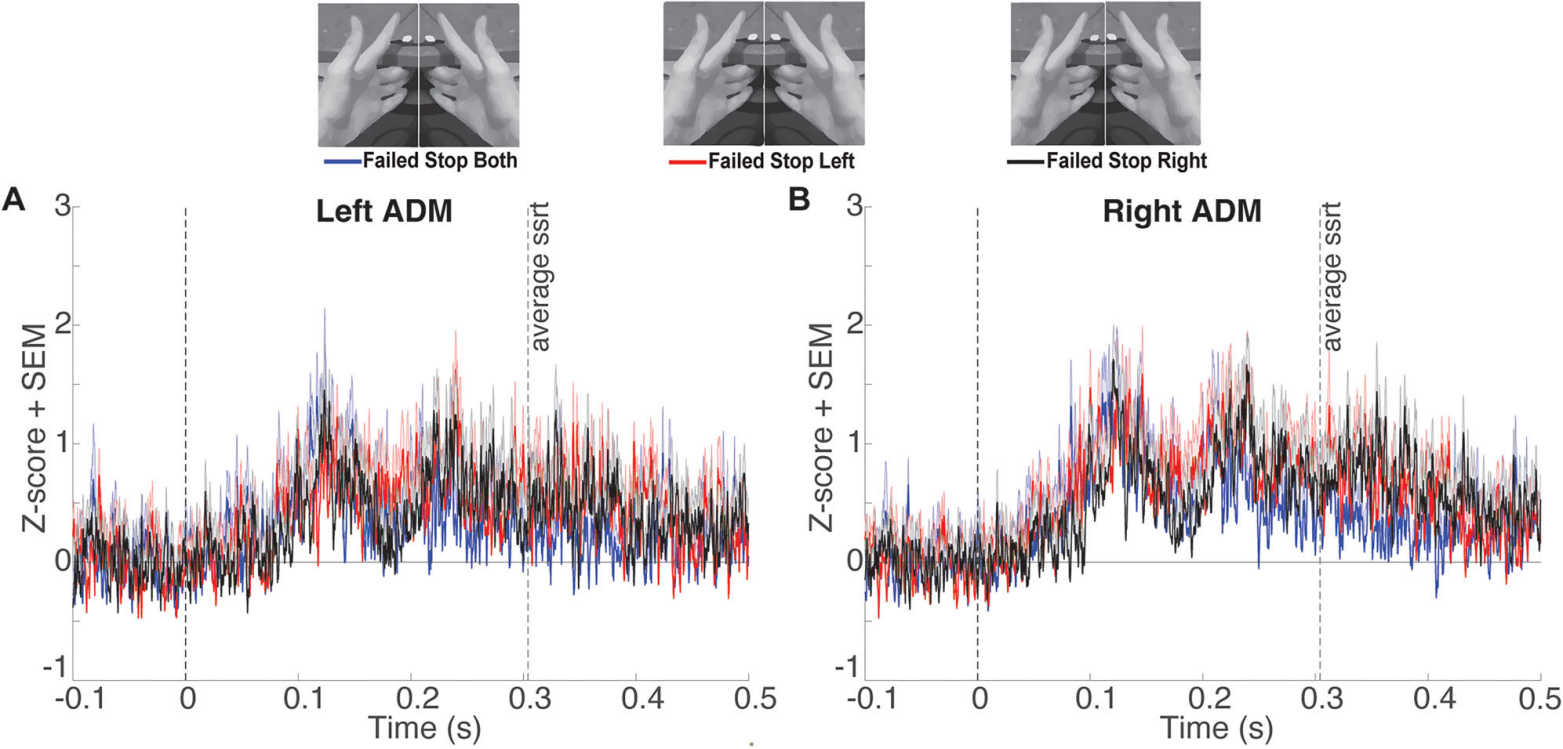
***A***, *Z*-scored tonic left ADM EMG amplitude (mean + SEM) comparing failed stop trials of all types locked to the stop signal at time point zero. Mean SSRT is denoted by the rightmost vertical dashed line. ***B***, Same as ***A*** for the right ADM. FDR-corrected comparisons (*p* *<* 0.05) were performed, but showed no significant differences between trial types at any time point.

Our design permitted us to compare between three types of failed stop trials: stop-both, selective stop-left, and selective stop-right. The contrast across these conditions is unique because the behavioral outcomes are matched, i.e., both index fingers are lifted in all three cases, and only the context differs. Differences in the pattern of tonic EMG between the bimanual and selective unimanual failed stop trials could suggest the operation of different inhibitory mechanisms. However, after FDR correction, there were no time points identified in which tonic ADM EMG amplitudes differed across the three types of failed stop trials (Fig. 7).

## Discussion

We analyzed the EMG of tonically contracting, nonresponding muscles during a selective ARI task to identify a continuous and temporally sensitive measure of global motor inhibition. We discovered a transient period of tonic EMG suppression during failed stopping compared with going 150−203 ms following the stop signal. The timing of this pattern is consistent with the hypothesized downstream effects of the hyperdirect pathway for interrupting ongoing motor responses. Moreover, this pattern was highly consistent in both hands regardless of whether the stop signal was bimanual or selective (unimanual). This further supports the existence of a nonselective, potentially global, stopping mechanism that suppresses muscle activity for ∼50 ms.

Previous studies examined EMG characteristics of the stopping process in the absence of tonic EMG activity. Raud and Huster (2017) observed partial EMG bursts (referred to as “subthreshold EMG” in their paper) in the responding effector during successful stopping, indicating the EMG activity associated with the initiation of a response can be rapidly terminated. They interpreted the peak of these partial EMG bursts as a physiological marker of when the stop process reached the muscle, ∼147 ms after the stop signal during a reactive inhibition task and at 152 ms during a proactive inhibition task. Other subsequent studies used the partial EMG response peak to estimate the latency of the onset of inhibition, or “cancel time,” during nonselective (Jana et al., 2020; Raud et al., 2020a,b, 2022) and selective stopping (Wadsley et al., 2022b; Salomoni et al., 2023). The observed onset of inhibition in task-relevant muscles in these previous studies closely matches the onset of EMG suppression we observed during failed stopping in the current study.

Using the tonic EMG trace, we also estimated the release of inhibition based on the time at which EMG activity on failed stop trials returned to a level like go trials. The offset of this inhibition period has been more difficult to estimate in past studies. TMS studies of CME modulation during stopping often only focused on one or a handful of time points in which they reported evidence of global inhibition, many reporting MEP amplitudes decreases within the same time frame at which we observed EMG suppression (Badry et al., 2009; Macdonald et al., 2014; Cowie et al., 2016). While these studies reported MEP amplitude decreases as early as 140 ms (Coxon et al., 2006) and as late as 220 ms (Majid et al., 2012) following the stop signal, the differences between these values and our reported values are small and may reflect differences in the behavioral tasks or the influence of inhibition operating at the cortical versus muscular level.

Interestingly, nearly all past studies that reported transiently reduced CME during successful stop trials reported no reduction during time-matched stimulation on failed stop trials (Badry et al., 2009; Greenhouse et al., 2012, Majid et al., 2012), or nonsignificant reductions (Cai et al., 2012). This suggests tonic EMG is more sensitive than TMS to inhibition in the context of ongoing responses, at least in the case of failed stopping. Alternatively, the observed inhibition during failed stopping may be a product of our task design and the proximity of the tonically contracted ADM to the responding FDI. It is difficult to dissociate between these possibilities in the current data, and it is one limitation of the design. Future studies can assess whether the observed patterns are explained by proximity, response coupling, or both.

While we identified many time points with significantly lower ADM activation during successful stopping compared with go trials and failed stop trials, the patterns were less consistent and were not subjected to the same interval testing. We note there was a considerable reduction in the density of time points showing significant differences in EMG amplitude between successful and failed stopping 150–203 ms after the stop signal (Figs. 3*B*, 4*B*, 5*A,B*). This indicates tonic EMG amplitudes during failed stop trials were reduced to values in a range similar to those of successful stop trials. Notably, we were unsuccessful in previous attempts to measure markers of inhibition in tonic EMG using two versions of unimanual reactive stop signal tasks. Specifically, we did not observe fluctuations in tonic EMG in a nonresponding hand during the performance of simple or choice versions of a standard stop task (Fisher et al., 2023). We speculate this may have been due to the decoupling of the hands during those tasks, whereas the ARI task employed here encourages participants to couple their hands and may explain why inhibition was detectable in the tonic EMG in this study (MacDonald et al., 2021; Wadsley et al., 2022a).

Global motor inhibition is hypothesized to be governed by the hyperdirect pathway (Aron and Poldrack, 2006) and is strongly implicated in reactive stopping (Chen et al., 2020). Historically, behavioral stopping has been characterized as a horse race between a go and stop process, with the faster process determining the behavioral outcome (Logan et al., 1984). More recent analyses suggest stopping is a two-part process in which a nonselective pause globally inhibits the motor system, followed by a selective cancel phase, which revises or completely cancels active motor programs. In rodents (Schmidt and Berke, 2017) and humans (Diesburg and Wessel, 2021), the pause phase is theorized to be initiated by the hyperdirect pathway, while the slower indirect pathway supports the cancel phase. The pause phase is triggered following all “salient” stimuli including the stop signal, resulting in transient inhibition during successful as well as failed stopping. Corroborative physiological evidence suggests a global pause mechanism is engaged during the early parts of both selective and nonselective stopping as well (Raud et al., 2020a; Wadsley et al., 2023).

While we did not find evidence of a two-phase pause-then-cancel signature (Wessel and Aron, 2015; Schmidt and Berke, 2017; Diesburg and Wessel, 2021) in our data, future studies can elucidate the potential separable influence of pause and cancel processes on tonic muscle activity by using a stimulus-selective response inhibition paradigm (Tatz et al., 2021; Wadsley et al., 2023). Alternatively, reanalysis of interactions between responding muscle and tonic muscle activity in the current data may provide insights into when each respective process engages.

Our approach offers several advantages over others that have been used to measure such markers of motor inhibition. EMG is noninvasive and is a less exclusionary option for assessing physiological mechanisms involved in stopping compared with TMS or MRI. EMG also possesses superior temporal resolution, and individual trial measurements of tonic EMG activity may provide further insights into trial-to-trial variability. This approach may be particularly appropriate for assessing hyperdirect pathway function in patients with basal ganglia dysfunction such as dystonia, chorea, ballism, or Parkinson’s disease.

### Limitations

EMG comes with its own set of constraints. The technique alone may be less sensitive than TMS-derived MEPs when there is no background muscle activity. Indeed, MEPs offer the ability to peek beneath resting EMG. This contrasts with EMG alone where it may be necessary to introduce background activity that is sufficient to reveal stopping-related suppression in the EMG trace. Furthermore, sustaining a tonic contraction in the ADM muscle may have influenced ARI task performance. However, our data are very similar to previous studies (Wadsley et al., 2023). While we did observe differences in the mean baseline RMS ADM EMG across blocks, this was unlikely to influence the data across our task conditions given that trial types were uniformly distributed across the task. Moreover, dynamic SSD staircasing would adjust for factors that could potentially interfere with the stopping process. Inhibition in the task-irrelevant ADM was not as apparent during successful as during failed stop trials likely because successful stop trials include a mixture of partial EMG responses and trials in which the EMG never initiates (Wadsley and Greenhouse, 2024). The tonic ADM EMG exhibited an apparent increase coupled to FDI response EMG onset during go and failed stop trials that was present but not as obvious for successful stop trials. This coupled increase in EMG was not anticipated but possibly exaggerated differences between going and failed stopping in our task. Differences early in the EMG epoch between successful and failed stop trials could arise from either inhibition or a lack of activation during successful stopping. Regardless, the presence of this response-coupled increase in ADM EMG activity may have unveiled inhibition that remained hidden during successful stop trials and may have provided sufficient EMG synchrony across trials for inhibition to be detectable, given the low likelihood of “trigger failures” and relatively narrow distribution of onset times. Relatedly, failed stopping across all trial types (stop-both, stop-left, and stop-right) involved bimanual responses resulting in similar EMG patterns. We only measured intrinsic hand muscles, and future studies can examine this effect in coupled muscles further from primary responding effectors to evaluate the spatial spread of inhibition in tonic EMG. Future studies can also evaluate whether our results generalize to other stop tasks such as a unimanual ARI task.

## Conclusion

In this study, we observed an electromyographic marker of inhibition acting at the periphery in a nonresponding muscle during action stopping. This measure can provide unique insight into the onset timing, magnitude, spatial spread, and duration of the underlying neural mechanisms. Our data are consistent with the engagement of a global “pause” mechanism that inhibits muscle activity starting ∼150 ms after the stop signal and persisting for ∼50 ms. This finding has immediate relevance to other studies of response inhibition and other behaviors associated with widespread transient motor system inhibition (e.g., detection of unexpected events). Our findings may be useful in contextualizing abnormalities in the dynamics of inhibitory processes acting on the motor system, and the noninvasive nature of our approach lends itself to applications in a variety of clinical and healthy populations.

## Synthesis

Reviewing Editor: James Howard, Brandeis University Decisions are customarily a result of the Reviewing Editor and the peer reviewers coming together and discussing their recommendations until a consensus is reached. When revisions are invited, a fact-based synthesis statement explaining their decision and outlining what is needed to prepare a revision will be listed below. The following reviewer(s) agreed to reveal their identity: Sumitash Jana, Ulrike Krämer. Note: If this manuscript was transferred from JNeurosci and a decision was made to accept the manuscript without peer review, a brief statement to this effect will instead be what is listed below.

The study by Mills et al. provides evidence that stopping responses can transiently reduce tonic muscle activity even in muscles that are unrelated to the task. While such global inhibition mechanism has been demonstrated before using combined EMG and TMS measurements, this study shows that EMG measurements alone can provide a sensitive and highly time-resolved marker of neural inhibitory processes. The results are interesting and will be of relevant to those studying response inhibition. If this method is established, it will help address fundamental questions about response inhibition. The manuscript is well-written, methods and results are clearly presented and the results clearly interesting to researchers of response inhibition and cognitive control. Data and code are shared on OSF.

Major comments:

1. The authors discuss at several points that the results might depend on the specificities of the task design. Specifically, they argue that the effects at the non-responding hand in selective stop-trials might be due to the bimanual task requirements which led to a stronger "coupling" of the hands, as they were unsuccessful in two previous attempts in observing transient decrease in task-unrelated tonic muscle activity (lines 395ff). This reasoning is a bit hard to follow. Studies of global inhibition have reported that MEP of task-unrelated muscles reduce during unimanual stopping, speech stopping, saccade stopping (the authors have cited several such studies). So why might the signature of global inhibition be observed when participants do a bimanual task that required coupling of hands? The authors should discuss their interpretation in more detail.

2. The authors consider that the proximity of the tonically contracted (task-irrelevant) ADM to the task-relevant FDI might have influenced the results, especially in failed stop trials (lines 382). This may limit the generalizability of the study and the interpretation of the results as a "global inhibition signal". The authors should discuss these limitations in the manuscript.

3. All figures show an increase of tonic ADM EMG activity in go-trials, which is absent or reduced in stop-trials. As the authors note in the Limitations (lines 437ff), this increase was not anticipated and might have been due to some coupling between ADM and FDI. While the reduced increase in EMG activity can be interpreted as inhibition, could it alternatively be interpreted as a lack of (global) activation, which is observed in stop-trials? This possibility should be discussed.

4. The sample size is relatively modest (N=18). Given that EMG is a noisy signal, the authors should attempt a power analysis that puts into context the suitability of their sample size to reliably detect the observed effects in the current study. The authors should discuss any potential limitations of the findings based on this power analysis.

5. The authors first average across trial types and then z-score the EMG with respect to a baseline (lines 205-210). Hence, if for any unknown reason the baselines are different between the trial types then it would be reflected in the EMG. The authors should redo the analyses by first z-scoring with respect to the baseline of individual trials and then divide into trial types.

6. The baseline period is considered as 500 ms prior to the stop signal. Since the go signal is presented during this period, is this appropriate? The authors should redo the analyses after considering the fixation period or inter-trial interval as the baseline to confim that the go signal does not influence the baseline.

7. The authors should clarify if participants were able to maintain the same level of tonic contraction in the absence of any visual feedback. Did the baseline activity change across blocks? Were there any fatigue effects?

8. What was the intertrial interval? Did the authors compare if the ADM activity went down to baseline after every trial (at least visually it seems as if the activity is above baseline even 0.5 s after the stop)?

9. Since the FDI is tonically active because of pressing down the response button, how efficient was the detection of Cancel time detection?

10. The ADM activity in the successful stop trials seem to diverge from the go and failed stop trails ~60 ms after the stop signal. This would suggest that the 'pause' process kicks in quite early which is inconsistent with TMS-MEP studies which show MEP reduction somewhere around 140-150 ms. One possibility is that the dynamics of the pause if different between the partial EMG and no EMG trials. The authors should report results where the successful stop trials are divided into these two trial types. The authors should also present the FDI activity of the relevant trials in the figures. The authors could also consider dividing the participants into low vs. high trigger failures and observe the ADM activity. This would test if the pause process is affected by trigger failures.

11. The authors visually identify a second decrease in the ADM activity around 250-300 ms and consider this to be a 'cancel' process. If this is truly a possibility, then it should be tested statistically. Else the simpler interpretation is that the reduction in EMG is driven by a single but sustained process.

Minor comments:

1. A sampling rate of 5 kHz seems a bit high. The authors should discuss the specific reason for choosing this sampling rate, and consider whether the results would hold up at a down-sampled rate.

2. The authors emphasize that their method is particularly interesting for clinical studies. How robust are the observed effects in individual participants? A violin plot of the average difference in critical time-windows in different conditions could be shown to clarify this point. This would be helpful for future studies to get an estimate of the variability of this effect even among neurologically healthy individuals.

3. Readers may get a better understanding of the effect of global inhibition on task-unrelated muscle if the negative results from Fisher et al study are presented here in the supplementary materials.

4. At least in the successful stop trials, there seems to be a sustained reduced activity from 60-450 ms. The authors should discuss what these early and late differences might indicate.

5. A decrease in ADM activity is seen in both successful and failed stop trials. This is not reflected in the title. The authors could reconsider the title.

6. Lines 70-73: Since Jana &Murthy (2018) studied the mechanism of stopping of coupled and decoupled eye-hand movements, bimanual responses should be replaced by effectors.

7. The number of significant digits reported for p-values should be the same throughout the manuscript (e.g., lines 252, 254, etc.).

8. Lines 274-279: There is some formatting issue.

9. Line 274-275: Is it correct to mention 'high response accuracy' for failed stop trials since the correct response in these trials in not making a response? It might be better to phrase this in a different way.

10. Table 1: The lift accuracy in the Stop-Left and Stop-Right is low. What might be the reason for this?

11. Line 292 / Table 2: It would help if the results were mentioned in the text.

12. Line 432: 'study' instead of 'studies'.

## Author Response

Synthesis of Reviews:

Synthesis Statement for Author (Required):

The study by Mills et al. provides evidence that stopping responses can transiently reduce tonic muscle activity even in muscles that are unrelated to the task. While such global inhibition mechanism has been demonstrated before using combined EMG and TMS measurements, this study shows that EMG measurements alone can provide a sensitive and highly time-resolved marker of neural inhibitory processes. The results are interesting and will be of relevant to those studying response inhibition. If this method is established, it will help address fundamental questions about response inhibition. The manuscript is well-written, methods and results are clearly presented and the results clearly interesting to researchers of response inhibition and cognitive control. Data and code are shared on OSF.

- We are grateful to the reviewers for their thoughtful and very constructive comments.

Major comments:

1. The authors discuss at several points that the results might depend on the specificities of the task design. Specifically, they argue that the effects at the non-responding hand in selective stop-trials might be due to the bimanual task requirements which led to a stronger "coupling" of the hands, as they were unsuccessful in two previous attempts in observing transient decrease in task-unrelated tonic muscle activity (lines 395ff). This reasoning is a bit hard to follow. Studies of global inhibition have reported that MEP of task-unrelated muscles reduce during unimanual stopping, speech stopping, saccade stopping (the authors have cited several such studies). So why might the signature of global inhibition be observed when participants do a bimanual task that required coupling of hands? The authors should discuss their interpretation in more detail.

- Coupling between hands may not be necessary to detect a global inhibition signature. We did not attempt a unimanual ARI task, which may reveal the same patterns as we observed with the bimanual task. However, with a unimanual design we would not have been able to compare selective-stop with stop-both conditions in our experiment (the latter of which is a form of simple stopping like unimanual stop trials). Additionally, coupling between the hands has been demonstrated to engage a less selective (i.e., global) stop process compared to decoupled contexts (Wadsley et al., 2019; 2022). Thus while there is inevitably overlap, our perspective is that the coupling-related effect is distinct from global inhibition alone. As the reviewer points out, MEPs of task-unrelated muscles are reduced during unimanual stopping. Moreover, between-hand coupling in the current study likely synchronized the background activity and increased the EMG sensitivity to global modulatory patterns. Specifically, background EMG in the ADM may have been more synchronized due to the coupling between the FDI muscles, and this may have increased our sensitivity to modulation that affected the two hands. We have elaborated on the EMG constraints in the Discussion section to highlight the strengths and weaknesses of the two different approaches (lines 469-463): "EMG comes with its own set of constraints. The technique alone may be less sensitive than TMS-derived MEPs when there is no background muscle activity. Indeed, MEPs offer the ability to peek beneath resting EMG. Coupling responses between the hands as in the current study may be necessary to introduce background activity that is sufficient to reveal stopping-related suppression in the EMG trace." 2. The authors consider that the proximity of the tonically contracted (task-irrelevant) ADM to the task-relevant FDI might have influenced the results, especially in failed stop trials (lines 382). This may limit the generalizability of the study and the interpretation of the results as a "global inhibition signal". The authors should discuss these limitations in the manuscript.

- Yes, proximity of the ADM may limit generalizability. We suggest future studies can examine other more distal muscles that have been coupled through response histories. We have expanded our discussion of this limitation (lines 415-413): "It is difficult to dissociate between these possibilities in the current data, and it is one limitation of the design. Future studies can assess whether the observed patterns are explained by proximity, response coupling, or both." 3. All figures show an increase of tonic ADM EMG activity in go-trials, which is absent or reduced in stop-trials. As the authors note in the Limitations (lines 437ff), this increase was not anticipated and might have been due to some coupling between ADM and FDI. While the reduced increase in EMG activity can be interpreted as inhibition, could it alternatively be interpreted as a lack of (global) activation, which is observed in stop-trials? This possibility should be discussed.

- We have added this possibility to our discussion (lines 474-476:) "Differences early in the EMG epoch between Successful and Failed Stop trials could arise from either inhibition or a lack of activation during successful stopping." 4. The sample size is relatively modest (N=18). Given that EMG is a noisy signal, the authors should attempt a power analysis that puts into context the suitability of their sample size to reliably detect the observed effects in the current study. The authors should discuss any potential limitations of the findings based on this power analysis.

- Previous studies have analyzed EMG in similar contexts with as few as 10 participants (Jana et al., 2020). EMG is noisy at a single-trial level. However, the amplitude of EMG bursts relative to background activity in this study (and other previous studies) indicates that this measurement can be highly sensitive when time-locked to go and/or stop signals. We calculated power for our comparison of rectified EMG activity for failed stop-both vs go trials in the window (138-212 ms) following the stop signal (or mean SSD) and observed a power of .95. This is now included in our Results section (lines 330-332). Given the observed power in our sample, we recommend that future studies use a similar sample size. "The calculated power for this comparison averaged across hands was 0.95, indicating that our sample was sufficient for detecting a Cohen's d effect size of 0.93." 5. The authors first average across trial types and then z-score the EMG with respect to a baseline (lines 205-210). Hence, if for any unknown reason the baselines are different between the trial types then it would be reflected in the EMG. The authors should redo the analyses by first z-scoring with respect to the baseline of individual trials and then divide into trial types.

- We reanalyzed the data as recommended, and the picture did not change much. There are still prominent differences between Go and Failed stop trials in the task-irrelevant ADM background EMG across all task conditions. We did note that the pattern of two distinct phases of inhibition became less clear, and we have removed the discussion points related to this. We have revised our figures and Results accordingly and we provide a description of this analysis in the Methods (lines 204-206): "For each participant, ADM EMG data were rectified and z-scored on a trial-by-trial basis, and then averaged across trials for each trial type generating 1.8 s duration mean EMG traces for go, stop-both, stop-left, and stop-right trials" 6. The baseline period is considered as 500 ms prior to the stop signal. Since the go signal is presented during this period, is this appropriate? The authors should redo the analyses after considering the fixation period or inter-trial interval as the baseline to confim that the go signal does not influence the baseline.

- EMG activity in the responding FDI was not present during this epoch (see Table 2), so it is unlikely that the rising bar stimulus on its own would influence this comparison. We caution against referring to this stimulus as a go signal since the goal of the task is to synchronize the lift with the crossing of the target line, and our chosen baseline would be more akin to a preparatory cue phase. Nevertheless, the ADM activity could modulate in anticipation of a response, and therefore we compared mean RMS of ADM EMG between in-task baseline with an ITI baseline epoch (.9 to 1.0 s after the lift target). A paired t-test showed no significant difference between these epochs (p = 0.46), and a Bayesian test showed substantial evidence for the null (equivalent distributions) BF10 = 0.31.

- Additionally, we have provided further support for our chosen baseline to remain consistent with previous TMS studies that have explored this window of time in the context of the ARI (lines 207-211): "Past studies conducted using TMS have compared relevant MEPs to a baseline MEP at -600 ms relative to lift time (MacDonald et al., 2014) up to -175 ms relative to lift time (Cowie et al., 2016). In both studies, the anticipatory nature of the task was not assumed to affect the integrity of the chosen baseline." 7. The authors should clarify if participants were able to maintain the same level of tonic contraction in the absence of any visual feedback. Did the baseline activity change across blocks? Were there any fatigue effects? - We did observe a significant difference in RMS ADM EMG baseline activity across blocks, which we have included in our results (lines 303-313). This may represent a fatigue effect. However, regardless of fatigue, the trial types were randomly distributed across the task so fatigue is unlikely to explain observed the differences across conditions. "There was a significant difference in baseline mean RMS ADM EMG across blocks [F(8,17) = 24.4, p < 0.01, η2 = 0.59]. Post-hoc t-tests showed that baseline mean RMS ADM EMG was greater during block 1 than during block 2 (t = 4.99, p < 0.001), block 3 (t = 8.18, p < 0.001), block 4 (t = 9.05, p < 0.001), block 5 (t = 9.49 p < 0.001), block 6 (t = 9.91, p < 0.001), block 7 (t = 10.27 p < 0.001), block 8 (t = 10.45, p < 0.001), and block 9 (t = 10.52, p < 0.001). Baseline mean RMS ADM EMG was greater during block 2 than during block 4 (t = 4.08, p < 0.01), block 5 (t = 4.52, p < 0.001), block 6 (t = 4.94, p < 0.001), block 7 (t = 5.30, p < 0.001), block 8 (t = 5.49, p < 0.001), and block 9 (t = 5.55, p < 0.001). There was no difference in baseline mean RMS ADM EMG between any other blocks. This pattern is consistent with a general decrease in ADM EMG activity over the course of the experiment that leveled off around block 3." - We discuss this in our Limitations section (lines 465-467): "While we did observe differences in the mean baseline RMS ADM EMG across blocks, this was unlikely to influence the data across our task conditions given that trial types were uniformly distributed across the task." 8. What was the intertrial interval? Did the authors compare if the ADM activity went down to baseline after every trial (at least visually it seems as if the activity is above baseline even 0.5 s after the stop)? - The ITI was 1 second (line 200). ADM activity was the equivalent at the end of the ITI and the baseline (please see Comment 6).

9. Since the FDI is tonically active because of pressing down the response button, how efficient was the detection of Cancel time detection? - To clarify, the pressing of the button did not result in tonic FDI activation in the EMG and this muscle was only engaged as an abductor for lifting the finger off of the button during the response epoch.

10. The ADM activity in the successful stop trials seem to diverge from the go and failed stop trails ~60 ms after the stop signal. This would suggest that the 'pause' process kicks in quite early which is inconsistent with TMS-MEP studies which show MEP reduction somewhere around 140-150 ms. One possibility is that the dynamics of the pause if different between the partial EMG and no EMG trials. The authors should report results where the successful stop trials are divided into these two trial types. The authors should also present the FDI activity of the relevant trials in the figures. The authors could also consider dividing the participants into low vs. high trigger failures and observe the ADM activity. This would test if the pause process is affected by trigger failures.

- With the revised analysis (Comment 5), the onset of the inhibitory process appears to occur later, at approximately ~140 ms after the stop signal. This is more consistent with previous findings.

- Unfortunately, there were very few partial EMG trials (Stop-Both 4.78 {plus minus} 4.12, Stop-Left 4.06 {plus minus} 3.61, Stop-Right 4.44 {plus minus} 4.26), which we now report in the manuscript (lines 302-303), so we do not believe we are sufficiently powered to perform the proposed comparisons between partial and no EMG trials.

11. The authors visually identify a second decrease in the ADM activity around 250-300 ms and consider this to be a 'cancel' process. If this is truly a possibility, then it should be tested statistically. Else the simpler interpretation is that the reduction in EMG is driven by a single but sustained process.

- With the updated analysis, we no longer see evidence of two distinct phases of inhibition, and we have revised the corresponding text in the manuscript (line 443).

Minor comments:

1. A sampling rate of 5 kHz seems a bit high. The authors should discuss the specific reason for choosing this sampling rate, and consider whether the results would hold up at a down-sampled rate.

- This sampling rate is the default on our system, which we chose in case we would like to perform time-frequency analyses of our data in the future. We suspect the results would hold up at a down-sampled rate because of the relatively long duration of the inhibition epochs.

2. The authors emphasize that their method is particularly interesting for clinical studies. How robust are the observed effects in individual participants? A violin plot of the average difference in critical time-windows in different conditions could be shown to clarify this point. This would be helpful for future studies to get an estimate of the variability of this effect even among neurologically healthy individuals.

- We have included the suggested violin plots as a new Figure 6, and we have added the following to our Results (line 329-330): "The difference in ADM EMG activity between go and failed stop trials was present in the majority of participants (Figure 6)" 3. Readers may get a better understanding of the effect of global inhibition on task-unrelated muscle if the negative results from Fisher et al study are presented here in the supplementary materials.

- We have included a citation to a poster describing this work in the main text which is available through our laboratory website. We can include these results within the Supplementary materials if the reviewer thinks they would be helpful. However, the methods of those experiments differed from the current experiment in several ways that we feel would be important to include and would go beyond the scope of supplements.

4. At least in the successful stop trials, there seems to be a sustained reduced activity from 60-450 ms. The authors should discuss what these early and late differences might indicate.

- The previously observed pattern is not as robust or consistent using our revised analysis approach, and this analysis no longer seems appropriate.

5. A decrease in ADM activity is seen in both successful and failed stop trials. This is not reflected in the title. The authors could reconsider the title.

- While we agree ADM activity decreases are observed for successful trials as well, the transient nature of the decrease in ADM activity is specific to the failed stop trials. Therefore, we prefer to leave the title as it was.

6. Lines 70-73: Since Jana &Murthy (2018) studied the mechanism of stopping of coupled and decoupled eye-hand movements, bimanual responses should be replaced by effectors.

- Replaced with effectors 7. The number of significant digits reported for p-values should be the same throughout the manuscript (e.g., lines 252, 254, etc.).

- Thanks. We have ensured these are consistent throughout.

8. Lines 274-279: There is some formatting issue.

- Thanks. We have fixed the formatting issue.

9. Line 274-275: Is it correct to mention 'high response accuracy' for failed stop trials since the correct response in these trials in not making a response? It might be better to phrase this in a different way.

- We agree this was confusing and have rephrased this.

10. Table 1: The lift accuracy in the Stop-Left and Stop-Right is low. What might be the reason for this? - Lift accuracy had a limited window of 100 ms and the feedback we provided, and the relatively large interference effect accounted for this.

11. Line 292 / Table 2: It would help if the results were mentioned in the text.

- We refer to the results in Table 2 (line 300-302).

12. Line 432: 'study' instead of 'studies'.

- Corrected
